# The Effect of the Molecular Architecture on the Antioxidant Properties of Chitosan Gallate

**DOI:** 10.3390/md14050095

**Published:** 2016-05-13

**Authors:** Chunhua Wu, Liping Wang, Zhongxiang Fang, Yaqin Hu, Shiguo Chen, Tatsuya Sugawara, Xingqian Ye

**Affiliations:** 1 College of Biosystems Engineering and Food Science, Fuli Institute of Food Science, Zhejiang Key Laboratory for Agro-Food Processing, Zhejiang R & D Center for Food Technology and Equipment, Zhejiang University, Hangzhou 310058, China; chwu0283@163.com (C.W.); zacamille@163.com (L.W.); chenshiguo210@163.com (S.C.); psu@zju.edu.cn (X.Y.); 2 Division of Applied Biosciences, Graduate School of Agriculture, Kyoto University, Kyoto 6068502, Japan; sugawara@kais.kyoto-u.ac.jp; 3 Faculty of Veterinary and Agricultural Sciences, the University of Melbourne, Parkville, Victoria 3010, Australia; zhongxiang.fang@unimelb.edu.au

**Keywords:** chitosan gallate, molecular architecture, antioxidant activity, grafting, gallic acid

## Abstract

To elucidate the structure–antioxidant activity relationships of chitosan gallate (CG), a series of CG derivatives with different degrees of substitution (DS’s) and molecular weights (MWs) were synthesized from chitosan (CS) and gallic acid (GA) via a free radical graft reaction. A higher MW led to a lower DS of CG. The structures of CG were characterized by FT-IR and ^1^H NMR, and results showed that GA was mainly conjugated to the C-2 and C-6 positions of the CS chain. The antioxidant activity (the DPPH radical scavenging activity and reducing power) were enhanced with an increased DS and a decreased MW of CG. A correlation between antioxidant activities and the DS and MW of CG was also established. In addition, a suitable concentration (0~250 μg/mL) of CG with different MWs (32.78~489.32 kDa) and DS’s (0~92.89 mg·GAE/g CG) has no cytotoxicity. These results should provide a guideline to the application of CG derivatives in food or pharmacology industries.

## 1. Introduction

Free radicals closely associated with reactive oxygen species (ROS’s) can cause oxidative damage to tissue and organs in biological systems, which subsequently triggers many diseases and ailments in humans (e.g. aging, cardiovascular disease, ischemic injuries, and cancer) and food deterioration [1,2]. Antioxidants obtained from natural or synthetic compounds are able to reduce or retard the rate of oxidative damage caused by ROS’s in a system. With the increasing health consciousness of consumers, natural antioxidants isolated from plants, marine creatures, and microorganisms have gained great interest [3,4,5].

Chitosan (CS), the second most naturally abundant polysaccharide after cellulose, is a linear and natural cationic copolymer consisting of randomly distributed β-(1→4) linked *N*-acetyl-d-glucosamine (GlcNAc) and d-glucosamine (GlcN) units [6,7,8]. The unique structure of CS is produced by the deacetylation of chitin, naturally occurring biopolymers in the shells of insects, crustaceans (such as crabs and shrimp), and the cell walls of fungi [9,10]. Due to its nontoxic, biodegradable, biocompatible, and antioxidantive properties, CS and its derivatives have received wide attention as a functional biopolymer for diverse applications, such as pharmaceutical and food packaging material [11,12,13]. It is suggested that these functions are dependent upon not only their chemical structure (such as introducing water-soluble entities, hydrophilic moieties, bulky and hydrocarbon groups, *etc.*) but also the molecular size [4,5,14].

It was reported that CS has radical scavenging activity on the DPPH, superoxide, hydroxyl radicals, and carbon-centered free radicals [15]. However, CS is only soluble in a few dilute acid solutions, which limits its applications [6,7,16]. Furthermore, poor H-atom-donating ability of CS that leads to it has not been able to serve as a good chain breaking antioxidant [17,18]. To overcome these disadvantages, several natural antioxidative agents have been grafted to the CS chain to enhance its functional activities [7,19,20,21].

Gallic acid (GA) is a well-known natural phenolic acid with strong antioxidant activities extractable from plants, especially from green tea [22]. Grafting of GA to CS has already been accomplished via enzymatic grafting reactions (tyrosinase, laccase, or peroxidase) [23,24] and carbodiimide (EDC)-mediated ester reactions [20,25,26]. However, these methods are either time-consuming or contain toxic compounds that are unsuitable for use as food supplements or nutraceuticals. Compared with other conventional modifications, a H_2_O_2_/ascorbic acid (Vc) grafting reaction is an eco-friendly grafting procedure because toxic products are not generated, and it is possible to perform these reactions at room temperature to avoid degradation of antioxidants. Moreover, the preparation procedure is relatively simple in comparison to the above two methods [1,17,27,28]. However, it was noted that the CS MW plays a key role in designing copolymers, which has not been well elucidated in synthesized chitosan gallates (CGs) via redox pair systems in previous studies [17,28].

The molecular architecture information on the antioxidant properties of CG has never been discussed. In this study, CG derivatives with different MWs and degrees of substitution (DS’s) were prepared by a H_2_O_2_/Vc redox pair system, and the reaction conditions were optimized. The effect of molecular architecture (MW and DS) on the antioxidant properties of CG was also investigated in order to understand the structure-activity relationships of the CG antioxidant derivatives. The potential toxicity of the derivatives was determined against HepG2 cells, which could be a guideline to the application of CG derivatives in suitable industries.

## 2. Results and Discussion

### 2.1. Preparation of CG Derivative

**Figure 1:**
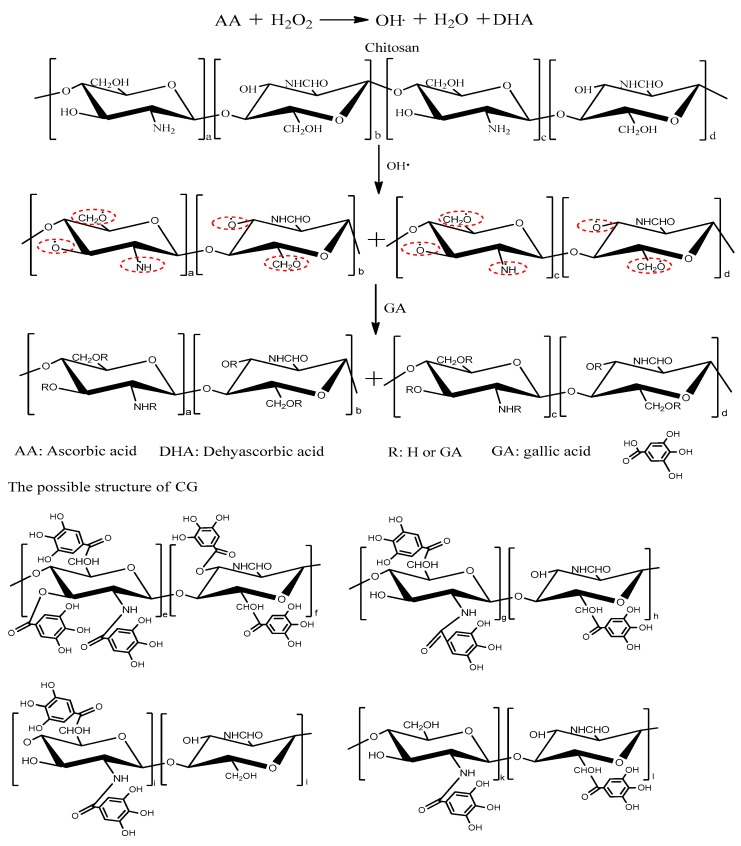
The proposed mechanisms for the synthesis of chitosan gallates (CGs) by free radical mediated graft copolymerization.

In this study, GA was successfully grafted onto CS chains by using a H_2_O_2_/Vc peroxide redox pair as radical initiators under nitrogen protection. The possible mechanism for the synthesis of CG derivatives is shown in Figure 1. The Hydroxyl radical (HO•) generated by the oxidation of Vc by H_2_O_2_ attacks H-atoms in R-methylene (CH_2_) or NH_2_ groups, hydroxyl groups (OH) of the hydroxymethylene group or of the CS chain, producing CS macro radicals [17,28]. Then, GA molecules that are in close vicinity of the reaction site become acceptors of CS macro radicals; thus, CG derivatives are formed [1,28]. Theoretically, the synthesis route is simple and does not generate toxic reaction products.

### 2.2. Effect of Reaction Conditions on the Degree of Substitution (DS)

#### 2.2.1. Effect of the Initial Ratio of GA:CS and the MW of CS on DS

**Figure 2:**
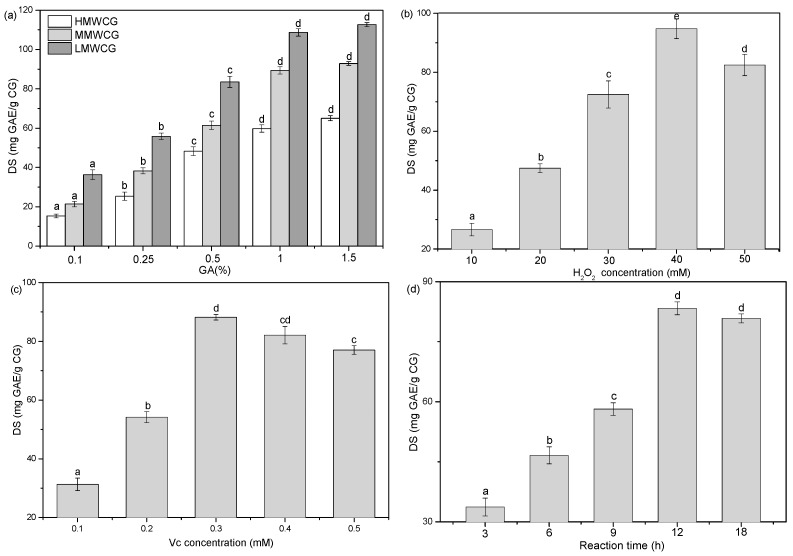
The effect of reaction conditions on degree of substitution (DS): (**a**) ratio of gallic acid (GA) to chitosan (CS) and molecular weight (MW) of CS on DS (*t* = 12 h, 20 mM H_2_O_2_, 0.3 mM Vc); (**b**) concentration of H_2_O_2_ on DS (MMWCG, *t* = 12 h, GA/CS = 1, 0.3 mM Vc); (**c**) concentration of Vc on DS (MMWCG, GA/CS = 1, 20 mM H_2_O_2_ and *t* = 12 h); and (**d**) reaction time on DS (MMWCG, GA/CS = 1, 20 mM H_2_O_2_ and 0.3 mM Vc).

As expected, the DS values of the CG samples increased with the increase of the GA:CS ratio (Figure 2a), which could be due to an accumulation of GA monomer molecules at the close proximity of the CS backbone [28]. However, at higher molar ratios, the DS did not increase correspondingly. The GA could be saturated in the system, or the reaction could have become a dynamic chemical equilibrium. Therefore, the further increase of the molar ratio showed no influence on the DS.

It is also noted that the DS clearly increased with the decrease of CS molecule weight. It was reported that the bioactivity of CS is strongly dependent on inter- and intra-molecular hydrogen bonds [14]. LMW CS has lower hydrogen bonds than M- and HMW CS; thus, LMW CS is prone to chemical modification [16]. The maximum DS was obtained in LMW CS-GA-1, which was 112.64 ± 1.03 mg·GAE/g CG.

#### 2.2.2. Effect of the Concentration of H_2_O_2_ and Vc on DS

As presented in Figure 2b, the DS increased from 26.62 to 94.81 mg·GAE/g CG at H_2_O_2_ concentrations between 10 mM and 40 mM, but then decreased to 82.49 mg·GAE/g CG over 40 mM H_2_O_2_. A similar trend was also observed within the concentration range of ascorbic acid (0.1–0.5 mM) (Figure 2c). It is well known that the Vc is easily oxidized by H_2_O_2_ and generates hydroxy radicals (HO•) [17]. The enhancement of H_2_O_2_ or Vc concentrations in the grafting system would produce more HO• free radicals. These radicals could further react with the CS backbones to form CS macro radicals. The more CS macro radicals formed, the higher the DS obtained. However, it was also suggested that the presence of too many HO• would stop the growing grafted chain by oxidative termination or degrade the CG molecular chain for more severe reaction conditions [28,29]. Therefore, the optimal H_2_O_2_ and Vc concentration in this grafting system was 40 mM and 0.3 mM, respectively.

#### 2.2.3. The Effect of Reaction Time on the DS

As shown in Figure 2d, the DS values of CG samples increased rapidly from 33.69 to 83.34 mg·GAE/g CG when the reaction time ranged from 3 to 12 h, and then decreased slightly between 12 and 18 h. Prolonging the reaction duration implied that there was more time on diffusion and absorption of the GA molecular to the active center of CS macro radicals, and more CG molecules were synthesized. The GA became saturated with CS macro radicals at 12 h, which could be the highest DS level. In addition, a longer reaction time would accelerate the degradation of CS or CG, which may be harmful to the final produce. Therefore, a further extension of reaction time did not increase the DS.

For further insight on the effect of the MW and DS on the antioxidant properties of CG, some CG derivatives were prepared according to the reaction conditions (based on the above factor analysis) in Table 1.

As shown in Table 1, to obtain similar DS’s of CG derivatives with varied MWs, three different MWs of CS were applied in the grafting reaction, according to the above factor analysis. A longer reaction time (15 h) and higher catalyst concentration led to a more serious degradation in the MW of CG; thus, a lower MW of a CG derivative was obtained. In addition, the different DS’s of CG derivatives were gained from the analysis in Section 2.2.1.

Based on the results of MWs and DS’s in various reaction conditions, it could be concluded that grafting and degradation occur simultaneously during the reaction process (as shown in Table 1). The degradation of CS molecules in other chemically modified treatments was also observed [30,31]. This may be attributed to the degradation effect of oxidation or free radicals on CS molecules [32,33]. However, as the reactions were conducted under a nitrogen atmosphere, which had excluded oxygen from these reactions, a free radical degradation process could have happened in this system. As depicted in Figure 1, a HO• is not only combined with CS to form macro radicals, but is also quickly pulled off a hydrogen atom from the CS chain and combines with it to form a water molecule which degrades the CS chain as follows:

$$\left(\text{GlcN}\right)m-\left(\text{GlcN}\right)n+\text{HO}•\to \left(\text{GlcN}\right)m+\left(\text{GlcN}\right)n+H_{2}O.$$

On the other hand, as the GA was covalently attached onto the CS backbone, the MW of the CG gradually increased with the increase of the DS, but not significantly (*p* > 0.05). The data from Table 1 also suggests that a series of CG samples has a narrow MW dispersity (lower PDI). These results demonstrated that estimating the MWs of CG products based upon the initial MW of chitosan could be misleading.

### 2.3. Characterization of CG

**Figure 3:**
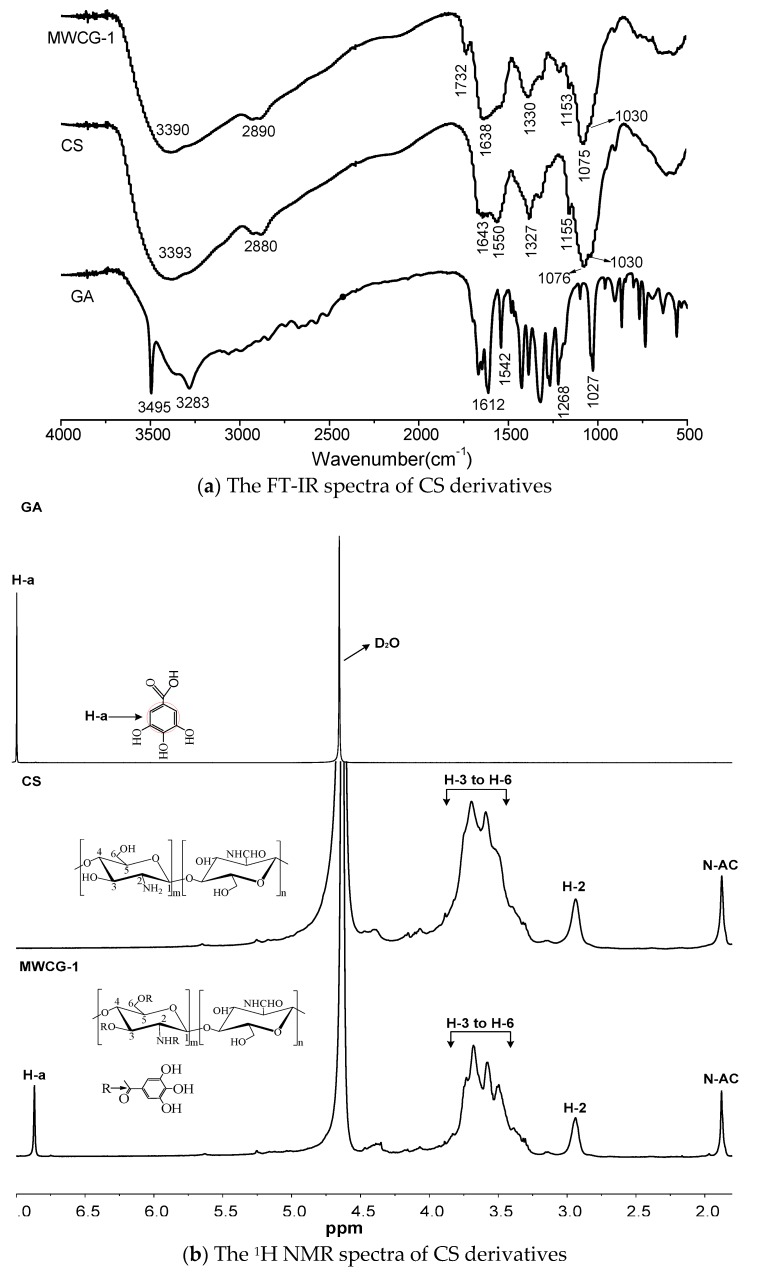

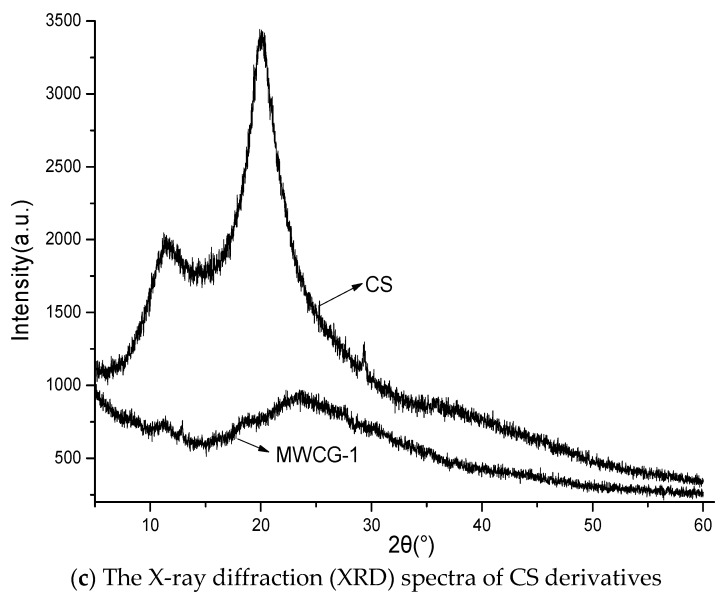
FT-IR spectra (**a**); ^1^H NMR (**b**) and XRD spectra (**c**) of CS derivatives.

In order to confirm the chemical structure of CG, the FT-IR spectra of samples were recorded. The main characteristic peaks of CS at 3393 cm^−1^ (O–H stretch), 2899 cm^−1^ (C–H stretch), 1550 cm^−1^ (N–H bend), 1327 cm^−1^ (C–N stretch) are shown in Figure 3a. There is a weak absorption peak of amide at 1643 cm^−1^ (representing C=O groups of amide), indicating a very high deacetylation degree of CS. In addition, three strong peaks at 1030, 1076, and 1155 cm^−1^, which were characteristic peaks of the saccharide structure, were also observed in the IR spectrum of CS [34,35]. Compared to the FT-IR spectrum of CS, the peak at 1550 cm^−1^ (N–H bending of the primary amine) of MWCG-1 was weaker, and the peak at 1638 cm^−1^ (C=O groups of amide) was enhanced, indicating that amide linkage between NH_2_ of CS and –COOH of GA were formed. In addition, a new peak at 1732 cm^−1^ corresponding to the C=O stretching of the carbonyl group was observed in MWCG-1 samples, suggesting the formation of a ester bond between –OH (at C-3 and/or C-6) of the CS chain and –COOH of GA. Due to the steric hindrance of C-3 position of pyranose ring, the possibility of substitution of GA at C-3 was very low [17,28]. Therefore, the gallyl group of GA most likely interacted with the active hydrogen of NH_2_ at C-2 (amide linkages) and the OH groups at C-6 position (ester linkages) of the CS chain. Similar results have been reported by Liu *et al.* (2013) [28], Cho *et al.* (2011) [1], and Spizzirri *et al.* (2010) [36].

The molecular structure of MWCG-1 was further confirmed by using ^1^H NMR spectroscopy. As shown in Figure 3b, the CS spectrum exhibits two typical signals at δ 2.96 and 1.88 ppm due to the H-2 proton of the GlcN and *N*-acetyl protons of GlcNAc, respectively; the multiplet at δ 4.51, δ 3.88–3.3 ppm are attributed to H-1 and H-3 to H-6 of the CS backbone. For the ^1^H NMR spectra of MWCG-1, it retains the characteristic signals of the parent CS; however, the chemical shifts of H-2, H-3, and H-6 of MWCG-1 were shifting to 2.86, 3.86, and 3.26 ppm, respectively, demonstrating that the substitution of GA occurred at positions C-3, C-6, or C-2. It was noted that a new signal appeared at 6.92 ppm (assigned to the phenyl protons of GA), which confirms the attachment of the phenyl group to the polymer chain. This result was consistent with that of others [1,28,36].

The crystallographic structure of MWCG-1 was determined by X-ray diffraction (XRD). As depicted in Figure 3c, two typical peaks were detected around 2θ = 10.4° and 22.1° in CS, which were assigned to crystal form I and crystal forms II, respectively. However, the XRD spectrum of MWCG-1 has much smaller peaks at around 2θ = 10.6° and 22.2°, confirming the interaction of CS with grafted GA. This result demonstrated that the incorporation of GA to the CS molecular chain reduced the crystallization of CS to some extent, suggesting that CS and GA chains were mixed well at a molecular level. This might be attributed to the fact that the intramolecular hydrogen bonding of CS had greatly decreased after grafting the GA group. As a result, the solubility of the MWCG-1 could be better than that of CS. Similar discussions for the changes of crystal structure and solubility of the CS derivatives have been found in the literature [26,28].

### 2.4. Antioxidant Assessments

#### 2.4.1. Effect of Molecular Weight on Antioxidant Activity

**Figure 4:**
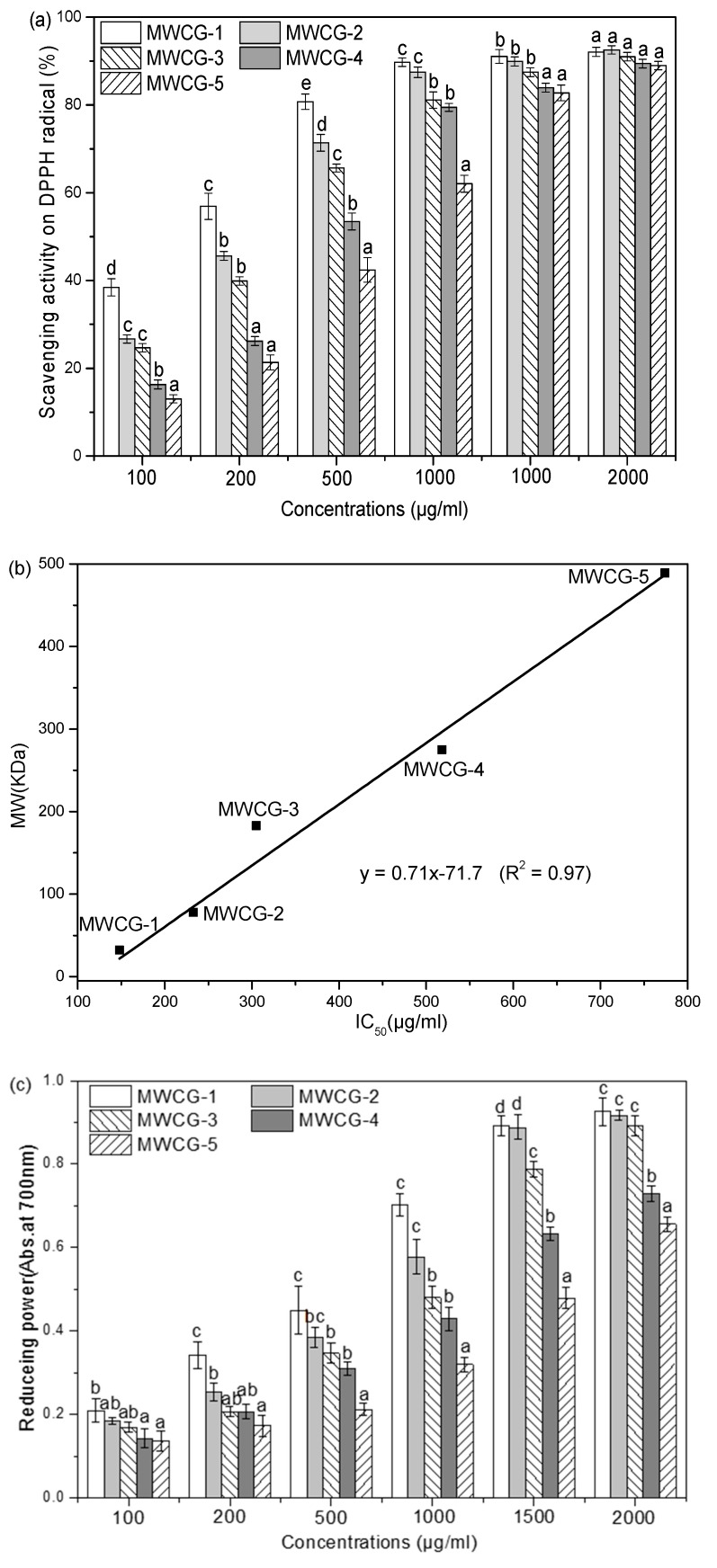

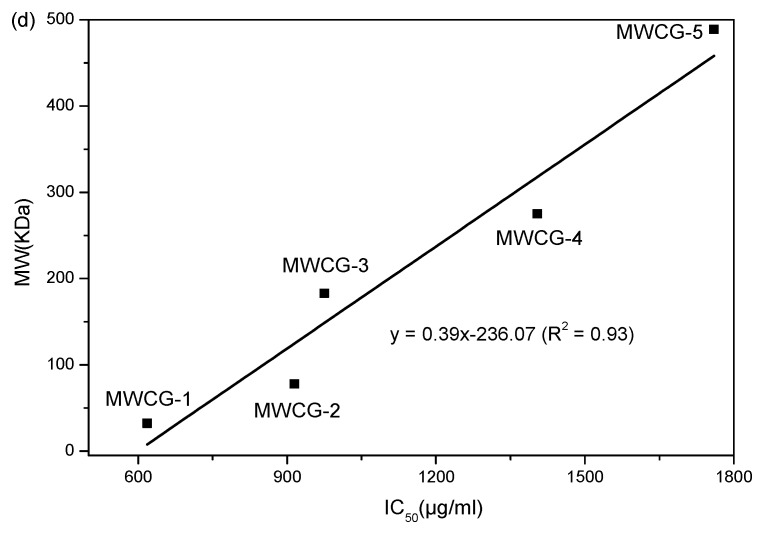
The effect of MW on antioxidant activity of CG samples. (**a**) Effect of MW and concentration on the DPPH scavenging activity of CG samples; (**b**) Relationship between MW and DPPH scavenging activity of CG samples; (**c**) Effect of MW and concentration on the reducing power of CG samples; (**d**) Relationship between MW and reducing power of CG samples.

As presented in Figure 4a, the scavenging activity of several different MW CG samples (100–2000 μg/mL) on DPPH radicals was significant and concentration-related. The scavenging rate of these CG samples increased with increasing concentration. The increase in concentration of CG resulted in the increase of total amine groups responsible for scavenging more radicals [37]. The IC50 values for MWCG-1, MWCG-2, MWCG-3, MWCG-4, and MWCG-5 were 148, 233, 305, 518, and 774 μg/mL, respectively, suggesting an inverse relationship between DPPH scavenging activity and the MW of CG (Figure 4b). The scavenging activities of LMW CG (MWCG-1 and MWCG-2) on DPPH radical were more pronounced than that of HMW CG (MWCG-4 and MWCG-5). The effect of the MW on CG scavenging activity might be attributed to the inter- and intra-molecular hydrogen bond of CS, which influences its biological activity [14]. CS has many hydrogen bonds on N2-O6 and O3-O5. HMW CG would have lower molecular mobility than the LMW CG, which would increase the possibility of inter- and intramolecular bonding among the HMW CG molecules [5]. Therefore, the chance of exposure of their amine, hydroxy, or GA groups might be restricted, which would have accounted for less radical-scavenging activity.

In terms of a reducing power test, the reducing power of CG samples correlated well with increasing concentrations and the change in reducing power for LMW CG (MWCG-1 and MWCG-2) were larger than that of HMW CG (MWCG-3 and MWCG-4) (Figure 4c). It indicates that LMW CG has a higher reducing power than that of HMW CG. Moreover, good positive correlations were observed between the reducing power and the MW of CG samples (Figure 4d), suggesting that CG with a lower MW would have relatively strong reducing power.

#### 2.4.2. Effect of the DS on Antioxidant Activity

**Figure 5:**
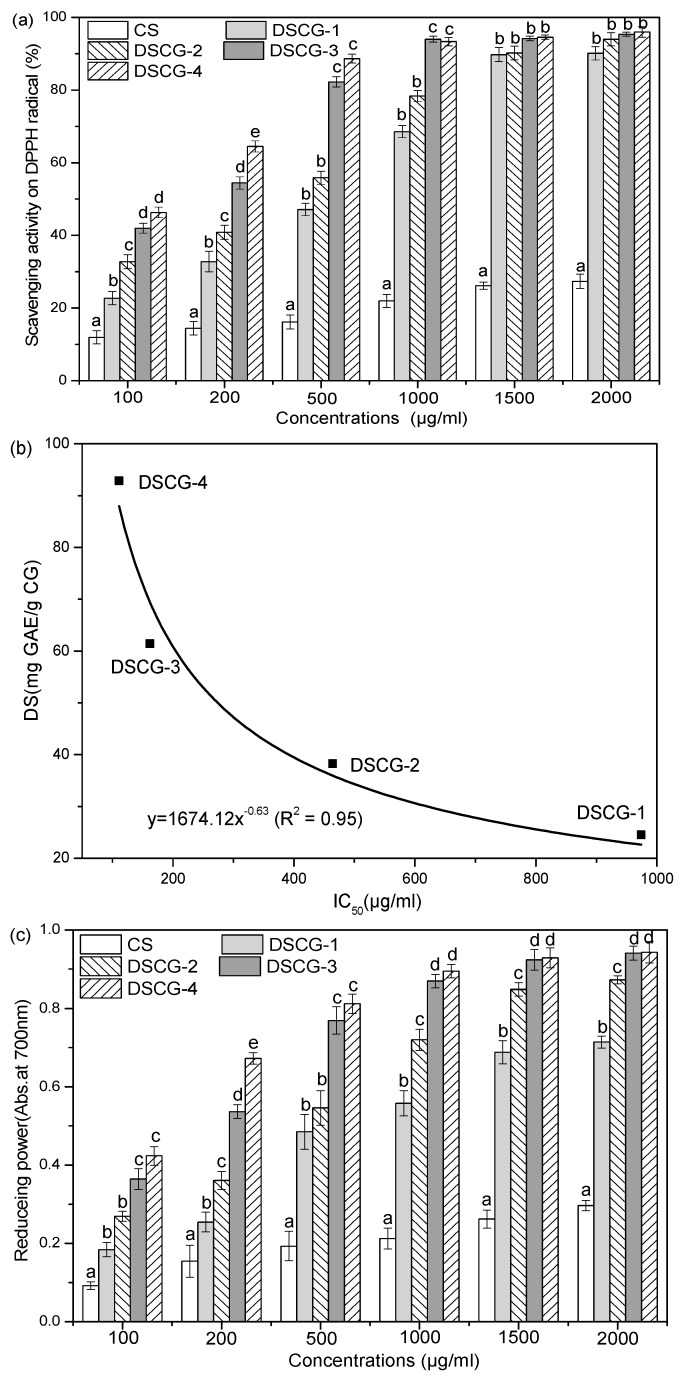

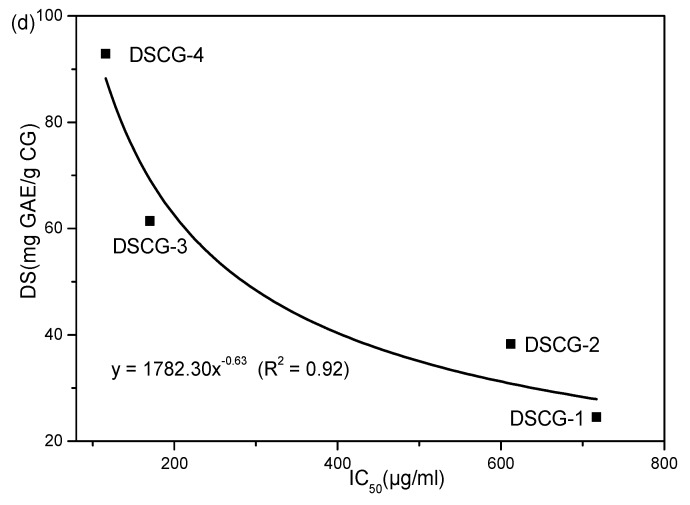
The effect of DS on antioxidant activity of CG samples. (**a**) Effect of DS and concentration on the DPPH scavenging activity of CG samples; (**b**) Relationship between DS and DPPH scavenging activity of CG samples; (**c**) Effect of DS and concentration on the reducing power of CG samples; (**d**) Relationship between DS and reducing power of CG samples.

As shown in Figure 5a, the DPPH scavenging activity of CS and CG samples was also a concentration-dependent manner. The scavenging rates increased with their increasing concentrations. IC_50_ of CG samples (111~945 μg/mL) were lower than that of CS samples (>2000 μg/mL). This indicated that CG may have higher activity upon the elimination of DPPH radical than the corresponding CS samples. Furthermore, good correlations were found between the radical-scavenging activity and the DS of CG samples (Figure 5b). As the DS of CG increased from 0 to 61.42 mg·GAE/g CG, the DPPH scavenging activity was enhanced significantly (*p* < 0.05). This phenomenon might be ascribed to the strong hydrogen-donating capacity of GA and -NH_2_ of CG. It is well-known that the antioxidants reduce the DPPH radical to a yellow-colored compound, diphenylpicrylhydrazine, and the extent of the reaction is dependent on the hydrogen-donating ability of the antioxidants [17]. The greater the dose of GA grafted on the CG chain, the higher the hydrogen-donating capacity, and thus the faster the scavenging on the DPPH radical. In addition, a lower DS resulted in more active amino groups in the CG chain. These active amino groups could also donate hydrogen to react with the DPPH radical [4]; therefore, a non-linear correlation between radical-scavenging activity and the DS of CG were observed.

The effect of the DS on reducing power of CS and CG samples are depicted in Figure 5c. The DS of CG showed a significant effect on reducing power activity that was proportionally increased by the GA content of CG. This suggested that the capacity of CG for reducing Fe^3+^ to Fe^2+^ was closely related to GA content. The reducing properties were generally associated with the presence of reductones, which have been shown to exert antioxidant action by breaking the free radical chain through the donation of a hydrogen atom [4,38]. Thus, the increased reducing power of the CG samples might be due to the excellent hydrogen-donating ability of the GA content. In addition, the reducing power of all types of CS and CG was correlated well with their increasing concentrations (Figure 5d).

Overall, the above results indicate that the antioxidant activity of the CG samples was closely related to their MW and DS and that the influence of the DS was greater than the MW.

### 2.5. Cytotoxicity Assessments

**Figure 6:**
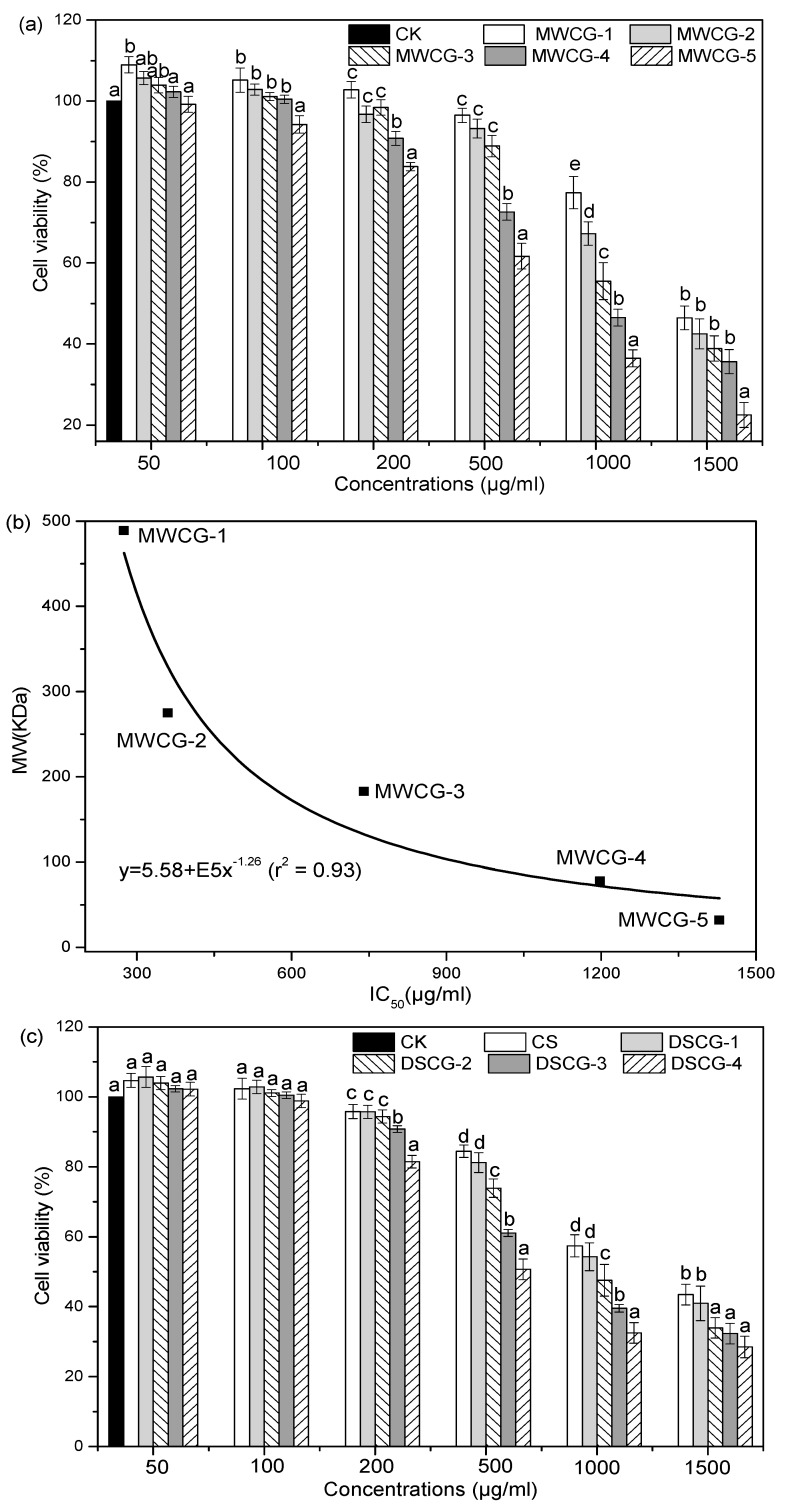

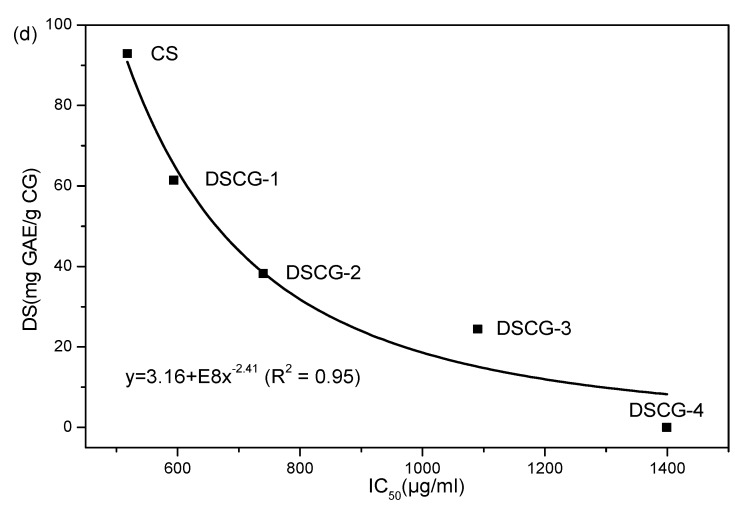
Cytotoxicity of the polymers at various concentrations.

MTT assays were performed to test the effects of CG copolymers on the metabolic activity of cells. As shown in Figure 6, the cytotoxicity of CG derivatives was dependent on its DS and MW. With the same DS and concentration, the cytotoxicity of CGs increased with its increasing MW. The compound of MWCG-5 was particularly toxic with an IC_50_ of 275 μg/mL, whereas MWCG-1 only exhibited cytotoxicity at a high concentration (1429 µg/mL). The influence of MW on the cytotoxicity of CG derivatives could be explained by the fact that the interaction of cationic molecules with plasma membranes increases with increasing MW, due to multiple attachments to cell surfaces [39]. A similar increase in cytotoxicity with increasing MW was observed for polylysine and poly (amidoamines) [40]. Moreover, an exponential relationship between MW and IC_50_ after 24-h incubation was established (Figure 6b), which could be used to predict the cytotoxicity of different MW CG derivatives.

The effect of the DS of CG derivatives on cell viability is presented in Figure 6c. The cytotoxicity of CG derivatives had no significant difference (*p* > 0.05) with a DS value of 21.37 and 38.25 mg·GAE/g CG at concentrations between 50 μg/mL and 200 μg/mL. However, as the DS further increased, the cell viability gradually decreased. A negative correlation was also observed between the cytotoxicity and the DS of CG samples (Figure 6d). In addition, the cell viability of CG derivatives was concentration-dependent. The higher concentration of CG derivatives with stronger cytotoxic effects might be due to the changes in osmotic pressure of the polymer solutions compared to cell culture medium [41].

## 3. Materials and Methods

### 3.1. Materials

Chitosan from shrimp with the MWs of 518.40 kDa, 211.59 kDa, and 98.67 kDa (coded as HMW-CS, MMW-CS, and LMW-CS, respectively), and a deacetylation degree of approximately 92%, was purchased from Qingdao Yunzhou Biochemistry Co. Ltd. (Qingdao, China). Chemicals of 2,2-diphenyl-1-picrylhydrazyl (DPPH), 3-(4,5-dimethylthiazol-2-yl)-2,5-diphenyltetrazolium bromide (MTT), gallic acid (GA), H_2_O_2_, Vc, Folin–Ciocalteu reagent, and D_2_O were purchased from Sigma Chemical Co. (St. Louis, MO, USA). All other reagents were of analytical grade.

### 3.2. Preparation of CG Derivative

The CG derivative was prepared by using a H_2_O_2_/ascorbic acid redox pair under a nitrogen atmosphere according to our previous study [42]. Briefly, the CS (1 g) was dissolved in 100 mL of 1% acetic acid (*v*/*v*) in 200 mL three-necked round bottom flask. Then, a certain amount of H_2_O_2_ and Vc was added into the reactor, and a slow stream of oxygen-free nitrogen gas was passed for 30 min with stirring. Afterwards, GA was added to the mixture at different molar ratios of the repeating unit of CS. The reaction was allowed to proceed at different CS MWs (98.67, 211.59, and 508.40 kDa), H_2_O_2_ concentrations (10, 20, 30, 40, and 50 mM), Vc concentrations (0.1, 0.2, 0.3, 0.4, and 0.5 mM), ratios of CS:GA (1:0.1, 1:0.25, 1:0.5, 1:1, and 1:1.5), and times (3, 6, 9, 12, and 18 h). The reaction was stopped by letting air into the reactor and then dialyzed with distilled water using an 8–14 kDa MW cut off membrane for 72 h to remove unreacted GA. Finally, the dialyzate was lyophilized to obtain a water-soluble CG derivative.

### 3.3. Characterization of CG Derivatives

Structural characterization of the blank CS and CG were performed by gel permeation chromatography-multiple-angle laser light scattering (GPC-MALLS), Fourier transform infrared spectroscopy (FT-IR), proton nuclear magnetic resonance (^1^H NMR) and X-ray diffraction (XRD) analysis. The molecular weights of the CS derivatives were analyzed by GPC with a MALLS detector (Dawn DSP, Wyatt Technology Corp., California, USA). All chitosan samples were dissolved in MQ water (5 mg/mL), filtered through a 0.22-mm syringe filter (Millipore Corp., Billerica, USA), and injected onto a TSK 3000 PWXL column. The samples were then eluted using 0.2 M ammonium acetate (pH 4.5) at a flow rate of 0.5 mL/min. The FT-IR was determined using an AVATAR 370 spectrophotometer (Thermo Nicolet Corporation, Madison, WI, USA) by scanning from 400 to 4000 cm^−1^. ^1^H NMR spectra were recorded at 25 °C with samples dissolved in CD_3_COOD/D_2_O (*v*/*v*, 1%) using a 600 MHz NMR spectrometer (Bruker Inc., Rheinstetten, Germany). The crystallographic structures of the CG derivatives were determined by a Bruker AXS D8 Advance X-ray diffractometer (Bruker Inc., Rheinstetten, Germany) using Ni-filtered Cu Kα radiation. The degree of substitution (expressed as the DS which is defined as the GA content in CG derivatives) was measured by the Folin–Ciocalteu method according to Liu *et al.* [28]. GA was used to calculate the standard curve, and the DS was expressed as milligrams of GAE per gram of the dry weight copolymer (mg·GAE/g CG).

### 3.4. Antioxidant Assessments

The antioxidant activities of CS and CG derivatives were evaluated using 2,2-diphenyl-1-picrylhydrazyl (DPPH) radical scavenging and reducing power assays.

The DPPH radical scavenging activity was estimated according to the previous method [20] with some modifications. Briefly, 200 μL of DPPH solution (0.4 mM DPPH in methanol) was mixed with 50 μL of samples (0.05–2 mg/mL) in a 96-well plate. The mixture was shaken vigorously and allowed to stand at room temperature for 0.5 h in the dark. Then, the absorbance of the mixture was measured at 517 nm by a microplate reader. The DPPH radical scavenging activity was calculated as followed:

$$\text{Scavenging activity}\left(\%\right)=\left[1-\frac{A_{1}-A_{2}}{A_{0}}\;\right]\times 100,$$

where A_0_ represents the absorbance of the control (water instead of sample), A_1_ represents the absorbance of the samples, and A_2_ represents the absorbance of the samples only (water instead of DPPH). The IC_50_ value was reported, which represents the concentration of the compounds that cause 50% inhibition of DPPH radical formation.

The reducing power was determined according to the previously described method [7] with some modifications. The reaction were carried out on 96-well plates, with each well containing a mixture of 50 μL of sample solution, sodium phosphate buffer (PBS, 0.2 M, pH 6.6), and K_3_Fe(CN)_6_ solution (1%, *w*/*v*), and were incubated at 50 °C for 20 min. After the addition of 50 μL of trichloroacetic acid (10%, *w*/*v*) and 30 μL of fresh FeCl_3_ (0.1%, *w*/*v*), the absorbance was measured at 700 nm. The IC_50_ value was reported, which represents the concentration of the compounds that generated 0.5 of absorbance.

### 3.5. Cytotoxicity Assessments

The cytotoxicity of CG derivatives was evaluated by a MTT assay using HepG2 cells according to our previous studies [43]. Cytotoxicity showing the cell viability rate was calculated by the following equation:

$$\text{Viable cell}\left(\%\right)=\frac{{\text{OD}}_{\text{samples}}}{{\text{OD}}_{\text{control}}}\times 100\%,$$

where OD_sample_ and OD_control_ were obtained in the presence or absence of CG derivatives, respectively.

### 3.6. Statistical Analysis

Analysis of variance was performed using ANOVA procedures of the IBM SSPS software (version 20.0, IBM Inc., Chicago, IL, USA). Duncan’s test was used to determine the difference of means, and *p* < 0.05 was considered to be statistically significant.

## 4. Conclusions

In this work, a series of antioxidant copolymers based on chitosan gallate were fabricated via a free radical graft reaction in a H_2_O_2_/Vc redox system. The effect of the molar ratio of GA to CS, the molecular weight (MW) of CS, the concentration of H_2_O_2_ and Vc, and the reaction time on degrees of substitution (DS’s) was investigated. The structures of CG were characterized by FT-IR, ^1^H NMR, and XRD, which showed that GA was conjugated to the C-2 and C-6 positions of the CS chain. However, GPC analysis indicated that the grafting reaction was accompanied with a degradation of the CS molecule. The antioxidant assay indicated that the molecular architecture (various MWs and DS’s) of CG samples had crucial effects on their DPPH radical scavenging activity and reducing power, and the influence of the DS on CG samples was greater than MW. In addition, the MTT assay showed no cytotoxicity for the CGs at a suitable concentration (0~250 μg/mL) with different MWs (32.78~489.32 kDa) and DS’s (0~92.89 mg·GAE/g CG). The results suggested that CG derivatives with a varying molecular architecture have the potential to be used as effective antioxidants in the pharmaceutical and food industries.

## Figures and Tables

**Table 1:** Physicochemical properties of CS derivatives.

Samples	CS		Reaction Conditions	DS (mg·GAE/g CG)	CG	
	MW/kDa	PDI			MW/kDa	PDI
MWCG-1	98.67 ± 4.15	1.82 ± 0.13	GA/CS = 0.5, *t* = 15 h, 40 mM H_2_O_2_, 0.4 mM Vc	73.21 ± 1.60	32.78 ± 1.35	1.61 ± 0.16
MWCG-2	98.67 ± 4.15	1.82 ± 0.13	GA/CS = 1, *t* = 12 h, 20 mM H_2_O_2_, 0.3 mM Vc	72.57 ± 2.02	78.37 ± 2.38	1.35 ± 0.08
MWCG-3	211.59 ± 6.89	1.90 ± 0.21	GA/CS = 1, *t* = 9 h, 20 mM H_2_O_2_, 0.3 mM Vc	72.93 ± 2.37	183.13 ± 3.27	1.42 ± 0.13
MWCG-4	508.40 ± 5.67	1.85 ± 0.14	GA/CS = 1, *t* = 15 h, 50 mM H_2_O_2_, 0.5 mM Vc	73.08 ± 2.17	275.92 ± 3.25	1.61 ± 0.14
MWCG-5	508.40 ± 5.67	1.85 ± 0.14	GA/CS = 1, *t* = 12 h, 25 mM H_2_O_2_, 0.3 mM Vc	74.33 ± 1.49	489.32 ± 4.62	1.57 ± 0.12
CS	211.59 ± 6.89	1.90 ± 0.21	GA/CS = 0, *t* = 12 h, 20 mM H_2_O_2_, 0.3 mM Vc	0	182.13 ± 3.27	1.44 ± 0.07
DSCG-1	211.59 ± 6.89	1.90 ± 0.21	GA/CS = 0.1, *t* = 12 h, 20 mM H_2_O_2_, 0.3 mM Vc	21.37 ± 1.26	184.46 ± 1.59	1.38 ± 0.14
DSCG-2	211.59 ± 6.89	1.90 ± 0.21	GA/CS = 0.25, *t* = 12 h, 20 mM H_2_O_2_, 0.3 mM Vc	38.25 ± 2.03	186.13 ± 3.27	1.45 ± 0.06
DSCG-3	211.59 ± 6.89	1.90 ± 0.21	GA/CS = 0.5, *t* = 12 h, 20 mM H_2_O_2_, 0.3 mM Vc	61.42 ± 2.16	188.89 ± 3.83	1.50 ± 0.19
DSCG-4	211.59 ± 6.89	1.90 ± 0.21	GA/CS = 1, *t* = 12 h, 20 mM H_2_O_2_, 0.3 mM Vc	92.89 ± 0.93	191.52 ± 2.64	1.43 ± 0.11

MW: Molecular Weight; PDI: Polydispersity Index.

## Abbreviations

The following abbreviations are used in this manuscript:

CS	Chitosan
GA	Gallic acid
CG	Chitosan gallate
MW	Molecular weight
PDI	Polydispersity Index
HMW-CS	High molecular weight chitosan
MMW-CS	Middle molecular weight chitosan
LMW-CS	Low molecular weight chitosan
DS	Degrees of substitution
HO•	Hydroxyl radical
MTT	3-(4,5-dimethylthiazol-2-yl)-2,5-diphenyltetrazolium bromide
DPPH	2,2-diphenyl-1-picrylhydrazyl
